# Innovations, implementation, and challenges of mobile health: a narrative review

**DOI:** 10.3389/fpubh.2026.1871930

**Published:** 2026-09-01

**Authors:** Xinyuan Li, Qin Huang

**Affiliations:** 1 Department of Gynecological Nursing, West China Second University Hospital, Sichuan University, Chengdu, Sichuan, China; 2 Key Laboratory of Birth Defects and Related Diseases of Women and Children (Sichuan University), Ministry of Education, Chengdu, Sichuan, China; 3 Department of Pathology, West China Second University Hospital, Sichuan University, Chengdu, Sichuan, China

**Keywords:** delivery of health care, disease management, health education, mobile applications, public health

## Abstract

Mobile health (mHealth) uses mobile communication technologies to provide new access to healthcare services and health information. This was a narrative review. This study aimed to review innovations, implementation, and challenges of mHealth, so as to investigate mHealth’s potentials and limitations in improving the efficacy of healthcare services, optimizing healthcare resource distribution, and improving population health outcomes. PubMed, Web of Science, Cochrane Library, and China National Knowledge Infrastructure (CNKI) were used to search mHealth-related literature published from 2015 to 2024. The application of health management software, such as MyFitnessPal and Health Buddy, in disease management, health education, and symptom monitoring was analyzed. Most of the included studies had small sample sizes or involved short-term interventions. The study results required validation through large-scale RCTs. mHealth faces challenges, such as technical compatibility, data security and privacy, user acceptance, cost issues, regulatory framework, network infrastructure, and clinical effectiveness. Most previous studies reviewing mHealth focus on a single disease or application of a single technology. This study examined the application of mHealth based on the World Health Organization’s global strategy on digital health and China’s national conditions. This study can provide a reference for mHealth policymakers and researchers.

## 1 Introduction

The Healthy China Initiative (2019–2030) is a development policy announced by the Chinese government in 2017. It sets out a grand blueprint, aiming to transform healthcare services from “treatment-centered” to “people’s health centered” through health promotion and education. As an important part of this transformation, mobile health (mHealth) uses mobile communication technologies, such as personal digital assistants, smartphones and wireless communications, to provide new access to healthcare services and health information. This emerging healthcare service mode can improve the accessibility and convenience of healthcare services and help to realize personalized medicine. A strategic approach utilizing the internet and new media is essential to achieve this goal. For example, the Chinese online health consultation platforms “Online Good Doctor” (“Hao Dai Fu Zai Xian” in Chinese) and “Dr. Chunyu” (“Chun Yu Yi Sheng” in Chinese) allow patients to communicate with doctors in real time through video calls and instant messages. This gives patients access to professional healthcare advice wherever they are. In addition, the official Weibo and WeChat accounts, such as “Healthy China” (an official Weibo account created by the National Health Commission of China), and “Dr. Dingxiang” (“Ding Xiang Yi Sheng” in Chinese, an official WeChat account created by DXY, a famous digital healthcare technology enterprise in China), regularly publish articles on disease prevention, along with lifestyle and healthcare tips. These are easy to share and spread quickly, greatly improving the public’s access to health information.

Meanwhile, government support is indispensable. The policy document “Opinions on Promoting the “Internet + Healthcare” issued by the State Council of China in 2008, puts forward the requirements, tasks and supporting measures for the development of “Internet + Healthcare” (1). The United States passed an important piece of legislation, the HITECH Act (Health Information Technology for Economic and Clinical Health Act), in 2009. As a part of the American Recovery and Reinvestment Act, the HITECH Act provides financial incentives for healthcare providers to use electronic health record systems to improve the efficiency and coordination of healthcare delivery (2). DeNicola et al. (3) systematically reviewed the effectiveness of telemedicine interventions in improving obstetric and gynecological health outcomes, demonstrating that the use of mHealth can significantly improve the quality of healthcare.

Despite the potential of mHealth, it also faces a series of challenges in its implementation. These challenges include technical compatibility, data security, privacy protection, user acceptance, cost issues, adaptability of regulatory frameworks, improvement of network infrastructure, and sufficient proof of clinical effectiveness. This study aimed to discuss these challenges and present solutions, providing a reference and suggestions for the development of mHealth.

Articles reviewing mHealth have been published in recent years. Most of these articles focus on a single disease, such as diabetes management (4) and mental disorders (5), or application of a single technology, such as remote electrocardiogram monitoring (6). This study examined the application of mHealth within the framework of the Healthy China Initiative, rather than being limited to a specific clinical specialty. It linked the World Health Organization (WHO)‘s global strategy on digital health with China’s national conditions, to explore the opportunities and obstacles in the localized implementation of the global strategy. This study reviewed the application of mHealth and made critical comments on the strength and limitations of the evidence for several applications. Greenhalgh et al. (7) highlighted that narrative reviews have unique value in integrating complex and heterogeneous evidence, especially suitable for comprehensive evaluations involving multiple disciplines and technologies. This is why this study was conducted using a narrative review method.

## 2 Methods

This article adopted a narrative review method to conduct a search and comprehensive review of literature on mHealth in health education. Narrative reviews differ from systematic reviews. Narrative reviews do not aim to conduct a rigorous synthesis of evidence for a specific clinical issue, but to conduct a panoramic review and critical analysis of research status, development trends, and key issues in a field (7, 8).

In this study, four databases—PubMed, Web of Science, Cochrane Library, and China National Knowledge Infrastructure (CNKI)—were used for literature search. The English search terms included “mobile health” OR “mHealth” AND “health education” OR “disease management” OR “remote monitoring” OR “digital health,” while the Chinese search terms included “mobile health care” (“Yi Dong Yi Liao” in Chinese) OR “mobile health” (“Yi Dong Jian Kang” in Chinese) AND “health education” (“Jian Kang Jiao Yu” in Chinese) OR “chronic disease management” (“Man Bing Guan Li” in Chinese) OR “remote monitoring” (“Yuan Cheng Jian Ce” in Chinese) OR “digital health” (“Shu Zi Jian Kang” in Chinese). The search period was from January 2015 to December 2024.

The inclusion criteria were as follows: ① a study involving the application of mHealth technology in health education, disease management, or health promotion; ② an original research, systematic review, or policy document issued by authoritative institutions; ③ published from 2015 and 2024; and ④ published in Chinese or English. The exclusion criteria were as follows: ① studies that involved traditional remote healthcare services, such as telephone consultations, without involving mobile technology; ② conference abstracts, letters, and editorials; or ③ studies without sufficient methodological information.

The strength of evidence for the included studies was evaluated based on the study design. Randomized controlled trials (RCTs) and systematic reviews/meta-analyses were considered to be of high quality. Cohort studies and case–control studies were considered to be of intermediate quality. Cross-sectional studies, case reports, and expert opinions were considered to be of low quality (9). For each included study, this study reviewed its clinical effect (if supported by RCTs or systematic reviews) and potential benefits (if supported by observational studies or theoretical analyses), and discussed its shortcomings in terms of user compliance, digital literacy, cost, and equity. Because evidence grading, such as the Grading of Recommendations, Assessment, Development and Evaluations (GRADE) framework, was not used in this narrative review, readers should judge the conclusions based on the specific evidence level.

## 3 Implementation of mHealth

mHealth is an important part of healthcare service innovation. The development and application of mHealth is being expanded in various aspects, such as remote patient monitoring, personal health management, management of chronic diseases, and responses to emergency medical services.

### 3.1 Remote patient monitoring

Remote patient monitoring uses mobile devices to collect patients’ physiological data, such as heart rate, blood pressure, and blood glucose levels. Yang et al. (6) established a data processing platform for 9,880 users wearing remote electrocardiogram monitors, using 3G or 4G for wireless data transmission. Dynamic electrocardiogram changes in users were monitored through various modules, such as automatic alarms, manual transmission, timed transmission, help transmission, expert attention, and alarm of electrode shedding, so as to achieve timely warning and detection of heart diseases. The results of Yang et al. have shown that the remote electrocardiogram monitors can provide early warning for the diagnosis of frequent premature ventricular beats, short atrial tachycardia, ventricular escape, malignant arrhythmia, acute myocardial ischemia, and nodoventricular escape. Early warning can provide more time for medical treatment and reduce patient death. Despite the study of Yang et al. had a large sample size (*n* = 9,880), it was a cross-sectional observational study without a randomized control group, making it difficult to avoid selection bias and confounding factors. Moreover, Yang et al. did not report the data concerning long-term compliance of the users. The impact of remote electrocardiogram monitoring on hard endpoints, such as cardiovascular mortality, needs to be validated by large-scale RCTs. In terms of popularization, the long-term wearing comfort and data transmission stability of wearable devices are the key factors associated with continuous use (10). The lack of the ability to operate smart devices may restrict their application among older adults and persons living in remote areas.

Diabetes management has become a hot issue in mHealth management. The internet-based glucose monitoring system (IBGMS) consists of three components: a mobile detection terminal, smartphone application software, and dedicated cloud platform, realizing the comprehensive management and service of personal health (4). In addition, several attempts have been made in China and other countries to introduce artificial intelligence (AI) technology into diabetes management. For example, “Kong Tang Bao,” an AI-based blood glucose control application in the Chinese language, is applied in management of chronic diseases and treatment; and “Song Yue,” an AI robot-assisted diagnosis system, can be used to diagnose diabetic retinopathy (4). Most IBGMS-related studies are small-sample before-and-after studies, which have indicated an improving trend in blood glucose management, but lacked data from large-scale RCTs. The accuracy of the AI-assisted diagnostic systems needs validation through multi-center and prospective studies. The deployment and maintenance costs of these systems are high, which may limit their promotion in primary healthcare institutions. The real-time upload of blood glucose data relies on a stable internet connection. The implementation effect of remote patient monitoring may be affected in rural areas with insufficient internet coverage.

### 3.2 Personal health management

mHealth is also applied in customized diet and exercise recommendations based on the user’s health data. MyFitnessPal is a popular health and fitness application that helps users achieve their health goals by tracking diet, exercise, and overall health. Its interface is simple and clear. Its design features include the ability to quickly add food by searching the food database or scanning a bar code, nutritional calculation, health advice, exercise tracking, and data synthesis analysis. It can facilitate communication and sharing of experiences between users. Hahn et al. conducted a qualitative study on the self-monitoring of female college students’ diet for one month through MyFitnessPal, and obtained research results from three aspects: ① Feelings. Negative emotions and increased concerns about weight and body image occurred; ② Cognition. More attention was paid to food choices and more time was spent on exercise; ③ Behaviors. Other forms of body self-monitoring were adopted, concerns about the healthiness of food increased, and the intake of unhealthy food was reduced (11). The study of Hahn et al. was a qualitative study with samples from female college students. The extrapolation of their results was limited. The impact of MyFitnessPal on objective health indicators, such as weight changes and improvement on body mass index, needs to be confirmed by quantitative studies. Previous studies have shown that mHealth applications had low long-term usage rates and many users stopped using the applications within a few weeks after downloading them (10, 12). Since these applications rely on users’ input of dietary data, their effects are limited for users with weak self-management intentions. The targeted population of these applications are those who have smartphones and health management awareness. This may exacerbate the inequality in access to healthcare resources.

### 3.3 Management of chronic diseases

Mobile applications are used to support patients’ disease self-management, with features such as medication reminders and symptom tracking. Naslund et al. reviewed 46 studies involving 12 countries, which showed that interventions of mHealth and electronic health played a significant role in disease self-management, relapse prevention, medication adherence and/or treatment, psycho-education, and symptom monitoring among patients with schizophrenia, schizoaffective disorder and bipolar disorder (5). Specific measures include ① Disease self-management and relapse prevention. Health Buddy, an automated home messaging application, plays a key role by displaying information concerning disease management. Users can report their symptoms through the application. The application provides feedback or recommendations based on the input information. It can regularly remind the user to have a symptom evaluation and can remotely transmit information to healthcare professionals. ② Promote medication and/or treatment. Short messages were used to remind patients to take medications. Electronic medicine bottle caps were used to record every opened bottle to monitor medication adherence. Wearable sensors were used to track ingestible digital tablets to track medication intake. ③ Psychological education. Smartphone applications and online social media platforms, such as HORYZONS, were used to provide psychological education and support social interactions. ④ Symptom monitoring. Smartphones and mobile platforms were used to automatically prompt participants to report symptoms at specific time of day, including mood, mental state, and positive and negative effects.

Most of the studies included in the study of Naslund et al. were pilot studies with a small sample size, with insufficient RCTs. Naslund et al. confirmed the differences in quality of evidence, with some studies having short intervention periods and high dropout rates. The cognitive impairment of individuals with severe mental disorders may affect their continuous use of mobile devices. They generally have a low level of digital skills and require additional training and support. The procurement and maintenance costs of smart devices may be barriers to the popularization of mHealTh services. The opportunities for individuals with severe mental disorders in low-income and developing countries to access mHealth services remain limited.

### 3.4 Response to emergency medical services

Mobile solutions are developed to rapidly respond to emergency medical services. In 2018, Hu et al. developed a heart rate monitor based on organic piezoelectric materials (13). The wearable device emits low noise and is comfortable to wear, thus improving its effectiveness in disease monitoring and early warning. HEARThermo is a watch-like monitoring device developed by researchers from National Cheng Kung University in Taiwan, China in 2020. It can measure the body surface temperature and heart rate every ten seconds, and can analyze the collected data by setting the algorithm of disease in the application matching the device to perform early warnings of the COVID-19 (9). These two studies were in the stage of technology development and proof-of-concept verification. They had not yet undergone large-scale clinical trials to validate their alert sensitivity and specificity. The study on HEARThermo was conducted using a small-scale test during the COVID-19 pandemic. Its ability to alert for other infectious diseases remains uncertain. In terms of industrialization, the cost of large-scale production of new sensor materials is high, and the production of wearable devices with disease alert functions may need to be registered under the medical device category and approved by relevant authorities, which inevitably increase the time and cost for product launch.

## 4 Enlightenment from the WHO’s strategy in mHealth

The WHO launched the “Global Strategy on Digital Health 2020–2025” in Geneva, Switzerland in 2019, setting out its vision, strategic objectives, guiding principles, framework for action and implementation plan. The overall goal is to improve human health through digital technologies and realize the vision of health for all (14). It emphasizes four strategic goals: ① promoting global cooperation; ② advancing universal health coverage; ③ protecting the health of the population during health emergencies; and ④ promoting health and well-being.

### 4.1 Building a national healthcare science and technology innovation system

The WHO recommends member countries establishing national digital health strategies and incorporating digital health into their national healthcare systems (14). China has made positive progress in this area. China’s 14th Five-Year National Health Plan proposes to promote the application of big data in health care and to coordinate the layout of national biomedical big data, biological sample resources and other platforms. However, the electronic health record systems used by different healthcare institutions vary and the data formats and standards are not unified, hindering the cross-institutional sharing of health information (15). Challenges to governance cannot be ignored. China’s laws and regulations on health data governance needs to be improved. Issues such as data ownership, usage boundaries, and responsibility entities need clarification (16). The acceptance and operational capabilities of staff from primary healthcare institutions towards digital health tools vary, hindering the implementation of relevant policies. A systematic capacity-building plan is imperative.

### 4.2 Developing and promoting appropriate health technologies

The WHO strategy emphasizes the use of digital platforms as a support to assist in establishing personal health profiles through AI and big data (14). China has made rapid progress in AI-assisted diagnosis and wearable health devices. However, the choice of appropriate technologies should match the conditions of different regions. AI-driven health management platforms have shown effectiveness in urban areas with well-developed network infrastructure. Technology solutions that overly rely on high-speed networks and smart terminals may not be applicable in rural and remote areas with weak network infrastructure. Many countries have faced controversies regarding data collection transparency and user informed consent in the promotion of digital health. Biases, privacy and security risks of AI in healthcare have also been widely discussed (17). This suggests that a data protection mechanism and technical ethics review framework need to be established simultaneously when promoting appropriate health technologies.

### 4.3 Promoting the application of big data in health care

The WHO strategy suggests member countries promoting an integrated national health information system covering the entire life cycle (14). China is continuously advancing the sharing of health big data based on regional population health information platforms, but data interoperability remains a key obstacle. China has not fully adopted unified health data exchange standards, such as HL7 FHIR, and there are technical barriers to data interoperability among the provinces (15). The secondary use of health big data, such as commercial research or insurance pricing, requires a clear regulatory framework to prevent data abuse. In addition, there is an urgent need to cultivate interdisciplinary talents who are proficient in both healthcare and digital technology. The WHO strategy also specifically points out that the construction of digital health human resources is a guarantee for implementation of mHealth (14).

### 4.4 Developing wearable health devices and AI-assisted diagnosis systems

The WHO strategy encourages member countries to utilize digital technologies to enrich health management approaches (14). China has made significant investments in wearable health devices and AI-assisted diagnostic systems, but there are several issues worth noting. The clinical effectiveness of AI-assisted diagnostic systems requires rigorous validation and regulatory approval. The United States Food and Drug Administration has issued guidelines for the regulation of mobile medical applications, classifying applications with diagnostic functions as medical devices for management (18). The differences in quality and standardization of data from wearable devices may affect their reliability in clinical decision-making. The data formats of devices from different manufacturers vary, making it difficult to integrate them into a unified health record system (15). Additionally, the principle of leaving no one behind, emphasized in the WHO strategy, requires attention to the accessibility of technology for older adults, the low-income population, and those with lower digital literacy (14, 19).

## 5 The role of third-party platforms in daily life

With the development of technology, third-party platforms, such as WeChat and Alipay, have become indispensable tools in Chinese people’s daily life. Their application in healthcare services is especially eye-catching. These platforms offer users unprecedented convenience by integrating mobile health capabilities, while also raising concerns about data confidentiality, informed consent, and data ownership.

### 5.1 Integration in health applications

Third-party platforms, such as WeChat and Alipay, allow health applications to access their huge user bases through open application programming interfaces and software development kits. Users can make appointments with doctors, consult on health issues, buy medicines, and receive remote healthcare services directly on these platforms. For example, WeChat users can easily make an appointment with a doctor and access online consultation services using the “City Service” module in WeChat. Alipay’s “Healthcare and Health” module offers similar services, including the use of an electronic healthcare insurance card and health record management.

### 5.2 Improvement in convenience

These third-party platforms have simplified the healthcare service workflow, enabling patients to access healthcare resources more quickly. For example, the “Future Hospital” module in Alipay enables patients to receive online services for the whole process from making an appointment with a doctor, to payment, to test report inquiries, thus reducing waiting time in hospitals. The “Smart Hospital” module in WeChat offers similar services, providing users with one-stop healthcare services through mini programs and WeChat public accounts.

### 5.3 Informed consent and data ownership

In terms of data management, third-party platforms encounter several ethical and legal issues. Users may not fully understand how their health data will be collected, stored, and used when they utilize the platform’s health management function. Previous studies have shown that the privacy policies of most health applications are lengthy and difficult to comprehend and users often agree to them without reading them (20). It is unclear in legal terms whether the health data generated by users on the platform belongs to the user personally, the platform, or a healthcare institution. China started to implement the Personal Information Protection Law in 2021. It classifies health information as sensitive personal information and requires obtaining the consent from the user before processing the data (21). Third-party platforms face challenges in ensuring that users are completely informed of the use of their data and personal information protection.

### 5.4 Commercial use of health date

The massive health data held by third-party platforms has significant commercial value and can be applied in advertising, health insurance pricing, and drug development and research. The commercial use of health data has raised concerns about the protection of patients’ rights. The European Union General Data Protection Regulation sets strict restrictions on the processing of health data, requiring data controllers to prove the legal evidence for their processing and provide transparent information to data subjects (22). In China, the Personal Information Protection Law and the Data Security Law provide a legal framework, but industry guidelines and regulatory rules are needed to clarify how third-party platforms can legally and compliantly utilize health data for commercial use.

### 5.5 Cybersecurity and user trust

With the digitization and centralization of health data, data confidentiality has become an important issue. Third-party platforms must ensure that users’ personal health information is secure against data breaches and unauthorized access. Health data breaches have occurred worldwide in recent years, undermining patient trust in digital health services (20). The third-party platforms need to strictly comply with data protection regulations, such as the European Union’s General Data Protection Regulation and China’s Cybersecurity Law. In addition, the platforms need to constantly update their security measures to address increasingly sophisticated cybersecurity threats. If users lose confidence in data security of the third-party platforms, it will seriously affect their willingness to use the platforms and the completeness of their health data.

### 5.6 Regulation of private information technology companies

Third-party platforms are created by private information technology companies. Their activities in the health sector require regulation. China’s regulatory framework for internet healthcare is improving. The National Health Commission of China has issued the Management Measures for Diagnosis and Treatment on the Internet, but there is still a need to strengthen supervision in health data governance, algorithm transparency, and third-party platforms’ responsibility boundaries. The United States Food and Drug Administration has established a grading regulatory system for mobile medical applications, taking different regulatory measures based on the risk level of application functions (18). China can draw on other countries’ experience to establish an mHealth product assessment and regulatory mechanism, including independent assessment of health applications and establishing algorithm review and data security audit systems.

### 5.7 Potential impact

The application of third-party platforms in the healthcare sector improves the accessibility and convenience of healthcare services and provides rich data sources for healthcare data analysis. This data can be used to optimize health care, predict disease trends, and make decisions on public health. However, this also requires the third-party platforms to protect the privacy of their users, ensure the transparency and legality of data use, and receive independent supervision and auditing.

## 6 Future directions

With the continuous development of 5G, AI, and other technologies, mHealth will be more convenient and efficient in the future. It also faces various challenges and restrictions:

① *Technical compatibility:* Compatibility between different devices and applications may be obstacles for the popularization of mHealth. The adoption of internationally recognized standard for health data exchange, such as HL7 FHIR, and the establishment of a unified data interface, are required to ensure seamless connection and sharing of information between different systems and platforms (15).
② *Data safety and privacy protection:* Collection and processing of sensitive health data by mHealth applications requires strong data protection measures to prevent data breaches and protect patient privacy. Data breaches can lead to severe consequences. Embedding data protection mechanisms (i.e., the privacy-by-design concept) during the system development is an effective approach to addressing this challenge (20). Relevant laws and regulations, such as the Health Insurance Portability and Accountability Act, need to be strictly complied with. Establishing a mechanism for health data security audits and penalties for violations are recommended.
③ *User acceptance and digital literacy:* Despite the convenience offered by mHealth, patients and healthcare staff have different levels of acceptance of new technologies. User awareness and trust in mHealth technologies need to be increased among patients and healthcare staff and relevant training is required. Digital literacy promotion programs are recommended to be launched, with particular attention paid to the older adults and low-income populations (19). The design of applications should focus on user experience, thereby reducing the operational barriers.
④ *Cost and sustainability*: Developing high-quality mHealth applications requires significant front-end investment, and there are additional costs associated with ongoing maintenance and updates. The return on investment may be uncertain, which may limit the widespread use of mHealth technology. Exploring diverse financing models, including government subsidies, health insurance payments, and public-private partnerships, and conducting health economics evaluations to provide evidence-based cost-effectiveness information, are key to promoting sustainable development.
⑤ *Regulatory framework and independent auditing:* The existing regulatory framework cannot keep up with the rapid development of mHealth. A dedicated mHealth product evaluation and supervision system is needed to independently assess and attest health applications (18). An algorithm review system is also needed to ensure the safety and effectiveness of AI-assisted diagnostic systems. Strengthening supervision and defining the responsibility boundaries of the third-party platforms are urgent tasks that need to be carried out. China can refer to the grading supervision pattern of the United States Food and Drug Administration and the European Union’s medical device regulations to establish a regulatory system suitable for China’s national conditions.
⑥ *Network infrastructure and health equity*: Use of mHealth applications may be restricted in areas with inadequate internet coverage. Investment in network infrastructure in remote areas needs to increase. Lightweight mHealth solutions suitable for low-bandwidth environments are recommended (19). Incorporating digital health equity into policy considerations and ensuring technological advancements not exacerbating the inequality in health resource distribution are inevitable parts of achieving the “Healthy China” goal.
⑦ *The accumulation of evidence for clinical effectiveness:* Despite the potential of mHealth, sufficient clinical evidence from clinical trials and studies is required to support the effectiveness of mHealth applications in different health settings and patients. Multi-center RCTs with large sample size and establishing a standardized index system for mHealth effect evaluation are recommended for promoting the sharing of research data, thereby supporting evidence synthesis (8). This is inevitable to improving the quality of evidence.

## 7 Conclusion

This study explored the application status, evidence strength, and challenges of mHealth in health education and healthcare services. Under the policy of Healthy China Action (2019–2030), mHealth is regarded as an important technological means for transformation of healthcare service patterns. By analyzing the applications, such as MyFitnessPal and Health Buddy, this study found mHealth’s potential in disease management, health education, and symptom monitoring. However, the high-quality evidence regarding the clinical effects of mHealth remains limited. Most of the studies included in this study are observational studies, small sample trials, or qualitative studies. The significant effects of some applications lack validation through large-scale randomized controlled trials and long-term follow-up studies. The assessment of the benefits of mHealth should be done with caution. The results of this study should be regarded as suggestive rather than definitive. More rigorous clinical trials and studies on implementation science are needed to confirm the effectiveness of mHealth.

With the development of 5G and artificial intelligence, mHealth is expected to become more convenient and efficient. Collaboration among multiple fields, such as healthcare, technology, and data science, and government support are required to formulate favorable policies and regulations. Increasing internet coverage (especially in remote areas) is crucial for ensuring the popularization and accessibility of mHealth services. mHealth is an emerging healthcare service mode. It has potential in improving the quality and efficiency of healthcare services and promoting personalized and precise healthcare. However, the evidence regarding its effectiveness is insufficient. More reliable clinical and implementation evidence is required to evaluate its effectiveness. By addressing key challenges, such as interoperability standards, privacy-by-design, independent assessment of applications, more effective regulatory supervision, and by fully leveraging technological advancements, mHealth is expected to make greater contributions to the modernization of healthcare services in the future.

## Data Availability

The original contributions presented in the study are included in the article/supplementary material, further inquiries can be directed to the corresponding author.
